# Cross-Disease Innate Gene Signature: Emerging Diversity and Abundance in RA Comparing to SLE and SSc

**DOI:** 10.1155/2019/3575803

**Published:** 2019-07-16

**Authors:** Anna Petrackova, Pavel Horak, Martin Radvansky, Martina Skacelova, Regina Fillerova, Milos Kudelka, Andrea Smrzova, Frantisek Mrazek, Eva Kriegova

**Affiliations:** ^1^Department of Immunology, Faculty of Medicine and Dentistry, Palacky University Olomouc and University Hospital, Olomouc, Czech Republic; ^2^Department of Internal Medicine III-Nephrology, Rheumatology and Endocrinology, Faculty of Medicine and Dentistry, Palacky University Olomouc and University Hospital, Olomouc, Czech Republic; ^3^Faculty of Electrical Engineering and Computer Science, Department of Computer Science, VSB-Technical University of Ostrava, Ostrava, Czech Republic

## Abstract

Overactivation of the innate immune system together with the impaired downstream pathway of type I interferon-responding genes is a hallmark of rheumatoid arthritis (RA), systemic lupus erythematosus (SLE), and systemic sclerosis (SSc). To date, limited data on the cross-disease innate gene signature exists among those diseases. We compared therefore an innate gene signature of Toll-like receptors (TLRs), seven key members of the interleukin (IL)1/IL1R family, and CXCL8/IL8 in peripheral blood mononuclear cells from well-defined patients with active stages of RA (*n* = 36, DAS28 ≥ 3.2), SLE (*n* = 28, SLEDAI > 6), and SSc (*n* = 22, revised EUSTAR index > 2.25). Emerging diversity and abundance of the innate signature in RA patients were detected: RA was characterized by the upregulation of *TLR3*, *TLR5*, *IL1RAP/IL1R3*, *IL18R1*, and *SIGIRR/IL1R8* when compared to SSc (*P*_corr_ < 0.02) and of *TLR2*, *TLR5*, and *SIGIRR/IL1R8* when compared to SLE (*P*_corr_ < 0.02). Applying the association rule analysis, six rules (combinations and expression of genes describing disease) were identified for RA (most frequently included high *TLR3* and/or *IL1RAP/IL1R3*) and three rules for SLE (low *IL1RN* and *IL18R1*) and SSc (low *TLR5* and *IL18R1*). This first cross-disease study identified emerging heterogeneity in the innate signature of RA patients with many upregulated innate genes compared to that of SLE and SSc.

## 1. Introduction

Rheumatoid arthritis (RA), systemic lupus erythematosus (SLE), and systemic sclerosis (SSc) are systemic autoimmune diseases characterized by overactivation of the innate immune system together with impaired downstream pathway of type I interferon- (IFN-) responding genes (IFN signature). Nevertheless, a certain heterogeneity in the IFN signature among those diseases has been recognized, and some patients even lack its presence [1–4].

Although the emerging role of the innate immunity in the pathogenesis of RA, SLE, and SSc has been demonstrated, there is no data on the cross-disease innate gene signature as well as its heterogeneity among those diseases yet. Numerous studies on individual innate immunity members in RA, SLE, and SSc showed the crucial role of Toll-like receptors (TLRs) and IL1 family [5, 6]. Notable examples of common innate pathways are (i) the involvement of the adapter protein MyD88 which is required for signal transduction by TLRs and receptors of the IL1 family, (ii) the activation of the type I IFN, and (iii) the presence of endogenous TLR ligands [7]. Besides shared innate pathways, disease-specific molecular and cellular mechanisms exist. In SLE, recent evidence has suggested a close relationship between the endosomal TLR activation and the disease onset [8, 9] with an essential role of endosomal TLRs in the generation of anti-nuclear antibodies and type I IFNs [10]. In RA, abundant activation of individual members of TLR and IL1 families was already evidenced with a proposed role for exogenous TLR ligands in the disease onset (i.e., *Proteus* infection of urinary tract, Epstein-Barr virus, and parvovirus B19) and for endogenous ligands in self-sustaining of the inflammatory loop [5, 11]. In SSc, signaling via TLR is increasingly recognized as a key player driving the persistent fibrotic response and is linked to the activity of TGF-*β*; however, the pathological role of TLRs and their ligands in SSc still remains unclear [12].

We undertook this study to elucidate the underlying differences in the innate immunity signature across three major autoimmune disorders using multivariate analysis. This first cross-disease analysis of the innate gene expression signature of 10 *TLRs*, 7 key members of the *IL1*/*IL1R* family, and interleukin 8 (*CXCL8*) in peripheral blood mononuclear cells (PBMC) from patients with active SLE, RA, and SSc revealed emerging diversity and abundance in RA compared to SLE and SSc. Our study contributes to further understanding of the innate signature underlying the immunopathology of major autoimmune diseases, with special emphases to discriminate shared and disease-specific expression patterns.

## 2. Materials and Methods

### 2.1. Study Subjects

The study cohort consisted of 86 Caucasian patients with autoimmune diseases from a single rheumatology center in Olomouc, Moravia region of Czech Republic. All enrolled RA/SLE/SSc patients met the 2010 ACR/EULAR classification criteria for RA [13], the ACR classification criteria for SLE [14], and the 2013 ACR/EULAR classification criteria for SSc, respectively [15]. To exclude heterogeneity due to the activity and inactivity of the diseases, only cases with active phenotypes of the disease classified according to common activity scores (Disease Activity Score in 28 joints (DAS28), SLE Disease Activity Index (SLEDAI), and revised European Scleroderma Trials and Research group (EUSTAR) index) were included: RA (*n* = 36, DAS28 ≥ 3.2), SLE (*n* = 28, SLEDAI > 6), and SSc (*n* = 22, revised EUSTAR index > 2.25).

The demographic and clinical features, used medication, duration of disease, and relative white blood count are described in Table 1. Distribution of lymphocyte, neutrophil, and monocyte counts did not differ between studied patient's groups (*P* > 0.05). The healthy control cohort consisted of 77 subjects (mean age 51 yrs, min-max 24-90 yrs, female/male 58/19) out of which were formed three age-/gender-matched groups for each disease: 63 controls for RA (mean age 56 yrs, min-max 41-90 yrs, female/male 45/18), 33 controls for SLE (40, 24-50, 27/6, respectively), and 48 controls for SSc (58, 48-90, 34/14, respectively). In all healthy subjects, presence of inflammatory autoimmune diseases in first or second degree relatives, recent vaccination, infection, and usage of immunosuppressive drugs were excluded by questionnaire.

The patients and control subjects provided written informed consent about the usage of peripheral blood for the purpose of this study, which was approved by the ethics committee of the University Hospital and Palacký University Olomouc.

### 2.2. Sample Processing and Real-Time Reverse Transcription-Polymerase Chain Reaction (qRT-PCR)

The PBMC were isolated from the peripheral blood collected in K_3_EDTA tubes by Ficoll density gradient centrifugation (Sigma-Aldrich, Germany) and stored in TRI Reagent (Sigma-Aldrich, Germany) at −80°C until analysis. Total RNA was extracted using a Direct-zol RNA kit (Zymo Research, USA) according to the manufacturer's recommendations. After reverse transcription with a Transcriptor First Strand cDNA Synthesis Kit (Roche, Switzerland), qPCR was performed in a 100 nl reaction volume containing a LightCycler 480 SYBR Green I Master mix (Roche, Switzerland) using a high-throughput SmartChip Real-Time-qPCR System (WaferGen, USA) as reported previously [16, 17]. Primer sequences are listed in Table S1 (Integrated DNA Technologies, USA). The relative mRNA expression was calculated using phosphoglycerate kinase 1 as a reference gene [18].

In order to assess the innate immunity gene expression pattern, the expression of *TLR* (*TLR1-10*), *IL1*/*IL1R* family (21 members), and *CXCL8* was investigated in PBMC. Based on pilot evaluation of qPCR assays on a cohort of 20 RA, 20 SLE, and 20 SSc patients, 14 assays of *IL1/IL1R* family members (*IL1A*, *IL36RN*, *IL36A*, *IL36B*, *IL36G*, *IL37*, *IL38*, *IL33*, *IL1R2*, *IL18RAP*, *IL1RL1*, *IL1RL2*, *IL1RAPL1*, and *IL1RAPL2*) were below the limit of detection of the system and thus excluded from further analysis. The study continued therefore by expression profiling of 18 innate immunity genes: *TLR1-10*, 7 members of the *IL1*/*IL1R* family together with *CXCL8*.

### 2.3. Statistical Analysis and Data-Mining Methods

Statistical analysis (Mann–Whitney *U* test, Benjamini-Hochberg correction) of relative gene expression values was performed using Genex (MultiD Analyses AB, Sweden) and GraphPad Prism 5.01 (GraphPad Software, USA). *P* value < 0.05 was considered as significant.

In this study, a set of multivariate data-mining analyses to visualize and characterize the gene expression heterogeneity between and within the diseases was applied. For a flowchart of the analysis process used, see Figure S1.

First, correlation networks using the LRNet algorithm [19] and Spearman's rank correlation coefficient were constructed and visualized to investigate the relationships between expressions of individual studied genes within the innate gene signature and to nominate the most representative molecules for the particular disease.

Second, Andrews curve analysis was applied for visualization of the structure in multidimensional expression data [20–23]. The relative gene expression values of individual patients were transformed using Andrews' formula (Equation S1); all calculations were performed by package Andrews from the R library [24]. The Andrews curves were plotted to visualize the differences between particular diseases using a set of significantly deregulated genes and the whole set of studied genes. The difference is demonstrated by separation of the Andrews curve's amplitudes and phase shift [20, 22, 23]. The curves of similar relative gene expression overlap between studied groups (Figure S2), while separation of curves demonstrates the differences in expression profiles (Figure S3) [20, 22, 23]. More detailed description of the Andrews curve analysis is stated in Supplementary File.

Third, we applied association rule mining, a technique for finding frequent patterns, correlations, or associations among the given data set [25] to investigate the heterogeneity within the diseases themselves. Firstly, each gene data set was divided into low/high expression groups by arithmetic means of relative gene expressions within the whole data set. The applied package “arules” in the R system [26] was used to extract rules (combinations of genes and its expression levels associated with the particular disease). Only a minimum number of top ranked rules describing the particular disease with a good confidence (threshold 0.75) and support were used.

## 3. Results

### 3.1. Innate Immune Gene Expression Pattern of RA, SLE, and SSc

In order to characterize innate immune signature in studied diseases, the expression profiles of selected innate immune genes between patients and healthy controls in all diseases were compared.

To exclude the influence of age on the gene expression, the healthy controls were subdivided into age-matched subgroups despite no differences being observed in the expression profile of all investigated genes in the formed subgroups (*P*_corr_ > 0.05). RA differed from controls by the upregulated expression of *TLR2*, *TLR3*, *TLR5*, *TLR8*, *IL1B*, *IL18*, *IL18R1*, *IL1RN*, *IL1RAP*, and *SIGIRR/IL1R8* (*P*_corr_ ≤ 0.05; Figure S4A, Table S2A). In patients treated with anti-TNF-*α* therapy, a trend to lower TLR5 levels in our RA patients was observed (*P* = 0.07). In SLE, downregulation of *TLR10* was observed when compared to healthy controls (*P* = 0.02); however, it did not reach significance after the correction for multiple comparisons (Figure S4B, Table S2B). SSc differed from controls by the upregulated expression of *IL1RN*, *IL18*, and *CXCL8* and downregulated expression of *IL1RAP* and *IL18R1* (*P*_corr_ ≤ 0.05; Figure S4C, Table S2C).

### 3.2. Cross-Disease Analysis of Innate Pattern in RA, SLE, and SSc

**Figure 1:**
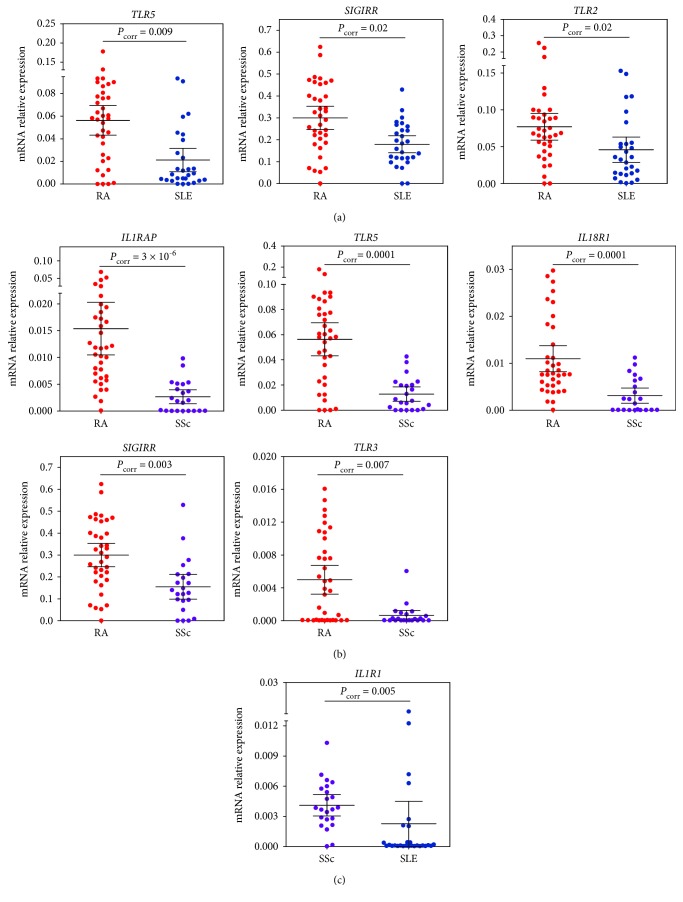
Relative mRNA expression levels of genes differentially expressed in (a) RA vs. SLE, (b) RA vs. SSc, and (c) SSc vs. SLE. Group means are indicated by horizontal bars; error bars indicate 95% CI.

To investigate the disease-specific innate immune gene expression pattern, we compared RA, SLE, and SSc patients to each other. RA differed from SLE and SSc by the upregulated expression of *TLR5* and *SIGIRR* (*P*_corr_ < 0.02; Figures 1(a) and 1(b), Tables 2(a) and 2(b), and Tables S3A and S3B). RA further differed from SLE by the upregulated expression of *TLR2* (*P*_corr_ = 0.02; Figure 1(a), Tables 2(a) and S3A) and from SSc by the upregulation of *TLR3*, *IL1RAP*, and *IL18R1* genes (*P*_corr_ < 0.007; Figure 1(b), Tables 2(b) and S3B). In SSc, the upregulated expression of *IL1R1* (*P*_corr_ = 0.005; Figure 1(c), Tables 2(c) and S3C) was observed when compared to SLE.

### 3.3. Visualization of Disease-Associated Gene Expression Pattern by Andrews Curves

**Figure 2:**
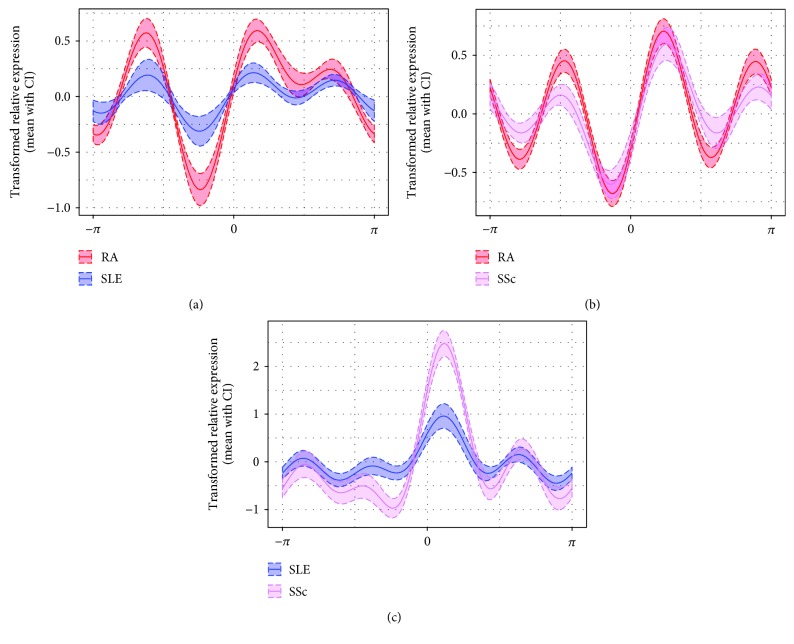
Differential innate gene expression analysis by Andrews curves between (a) RA vs. SLE, (b) RA vs. SSc, and (c) SLE vs. SSc—representative examples. The Andrews curves were calculated for various combinations of gene expression values from the whole set of studied genes. Examples show the results of the Andrews curve analysis for the combination of (a) *TLR3*, *TLR7*, *TLR8*, *IL1R1*, *IL1RN*, and *IL18R1*; (b) *TLR3*, *TLR4*, *TLR6*, *TLR10*, *IL1B*, *IL1R1*, and *SIGIRR*; and (c) *TLR4*, *TLR6*, *TLR7*, *TLR8*, *IL1R1*, *IL1RN*, and *IL18*. For those sets of genes, a good separation of diseases was observed as visualized by separation of the curve's amplitudes and phase shift. An example of combination of genes which does not discriminate between disease groups is shown in Figure S2. Full lines represent the mean values, the dashed lines 95% confidence intervals.

To investigate the disease-associated gene expression pattern, Andrews curves were used to visualize the differences between particular diseases using a set of significantly deregulated genes and the whole set of studied genes. First, we assessed the differences in the innate expression pattern of genes revealed by classical statistics. Although a good separation of Andrews curves on the basis of significant genes was observed (Figure S3), better separation of the studied diseases was obtained when a whole set of studied genes was used (Figure 2).

### 3.4. Innate Pattern Characteristics of RA, SLE, and SSc

Next, we applied the association rule analysis to identify rules (set of genes including their expression levels) describing a certain disease within the three studied diseases. Based on the results from the Andrews curves, association rule analysis was performed using the whole gene set.

**Figure 3:**
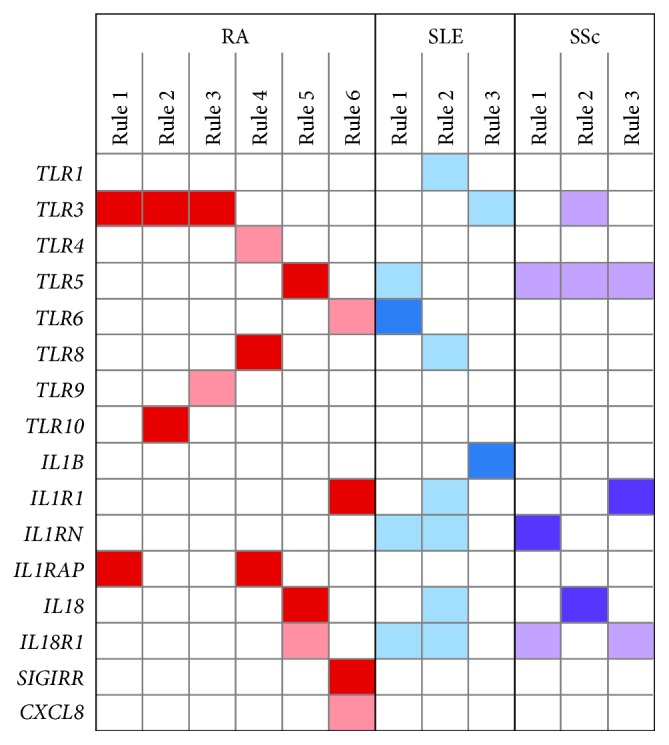
Association rules describing RA, SLE, and SSc. Association rule analysis revealed a minimum of six rules for RA, three rules for SLE, and three rules for SSc, able to discriminate among all studied diseases with the accuracy above 77%. Columns represent individual rules (combinations of genes and its expression levels characterizing the particular disease). Dark/light color means high/low gene expression levels (cut-off: mean gene expression of the whole data set).

For RA, six rules were identified, thus showing high heterogeneity within this group of patients when compared to SLE and SSc (Figure 3), where for each of them, three rules were identified. In RA, a high level of *TLR3* and *IL1RAP* mRNA was identified in three and two rules, respectively. In SLE, low expression levels of *IL1RN* and *IL18R1* appeared in two rules, and in SSc, a low level of *TLR5* and *IL18R1* mRNA occurred in three and two rules, respectively. The obtained association rules and their support and confidence values deciphered for RA, SLE, and SSc patients are listed in Table 3. The accuracy of classification by using these rules for RA, SLE, and SSc was 83%, 78%, and 77%, respectively. Comparison of rules for each disease revealed that *TLR3*, *TLR5*, *IL18*, *IL18R1*, and *IL1R1* genes occurred in rules for all studied diseases, showing good discriminant power among studied autoimmune diseases as visualized by the Andrews curves (Figure S5).

## 4. Discussion

This study focused on the innate immunity gene signature among major autoimmune diseases: RA, SLE, and SSc, showing heterogeneity in the innate signature among and within these diseases. This first cross-disease study showed the highest diversity and abundance in the innate signature in RA when compared to SLE and SSc.

Innate immunity plays a key role in the pathogenesis of autoimmune rheumatic diseases as evidenced from numerous studies on individual members of innate immunity pathways [5, 6]. However, little is known about the similarities and differences in the innate signature at the molecular level between and within these diseases. Therefore, we investigated the differential expression of key innate genes in RA, SLE, and SSc. Importantly, our study was restricted only to the cases with active disease in order to exclude heterogeneity due to the activity and inactivity of the diseases. To obtain a more complex picture, the multivariate analysis was applied to assess the complexity of the differential innate signature having an advantage over classical statistical approaches due to taking into account the intrinsic characteristics of gene expression data and assessing the relationships between studied molecules.

Firstly, we applied Andrews curve analysis for assessment of differences and similarities in the gene innate signature between studied diseases, an approach particularly useful for visualization of the structure in multidimensional data [20, 21]. When using combination of genes reaching statistical significance as well as using the whole gene set, we confirmed the diversity among innate profiles in RA, SLE, and SSc by Andrews curve analysis. Upregulated expression of *TLR3*, *TLR5*, and *SIGIRR* was characteristic for RA when compared to both SLE and SSc. An intracellular receptor TLR3 recognizing dsRNA has been shown to be involved in the RA pathogenesis: necrotic synovial fluid cells release RNA that can activate TLR3 in RA synovial fibroblasts [27]. TLR5, a surface receptor highly upregulated in our RA patients, recognizes bacterial flagellin. However, their endogenous ligand(s) in synovial fluid able to activate TLR5 in RA is(are) still unknown [28, 29]. In line with our results, increased TLR5 in peripheral blood myeloid cells correlated with RA disease activity and TNF-alpha levels [30]. There is also evidence that anti-TNF-*α* therapy markedly suppress TLR5 expression in RA monocytes [31], a trend which was also observed in our study. Also, the next highly upregulated SIGIRR (IL1R8/TIR8), an orphan receptor required for the anti-inflammatory effects of IL37, has been reported in RA synovial tissue previously [32].

Also, other genes such as *TLR2*, *IL1RAP*, and *IL18R1* from the differential innate signature associated with RA revealed by our analysis were reported in autoimmune conditions previously. In line with our results, abundant TLR2 on monocyte subsets in active RA produced a spectrum of proinflammatory cytokines after stimulation [33]. TLR2 recognizes a wide range of conserved microbial products, probably due to its cooperation with TLR1 or TLR6, as well as its hypothetic ligand HMGB1 released from dying and activated cells [34]. Regarding *IL1RAP* and *IL18R1*, their upregulated expression in RA was reported recently [16] and their downregulation in SSc we report here for the first time. Finally, SSc was characterized by an increase in *IL1R1* in comparison to SLE. The first evidence about critical involvement of IL1R1, an essential mediator for proinflammatory IL1 signaling [35], in fibrotic processes has been already reported in a murine lung injury model [36]. Importantly, data from our cross-disease analysis are in line with previous studies on individual innate members and basic statistical analysis and further highlight the activation of innate immunity in RA when compared to SLE and SSc. The infectious agents and endogenous ligands activating innate receptors leading to a self-sustaining inflammatory loop responsible for chronic and destructive progression in RA need to be further elucidated.

Next, we investigated the differential innate signature among and within the studied diseases by association rule analysis, a method commonly used to uncover the most frequently purchased combinations of items in a market basket analysis. It has been shown that this analysis is highly convenient for gene expression datasets [37, 38] and gives additional information due to preservation of the causality between the gene expression level and phenotype [37]. For RA, six rules were identified, thus showing high heterogeneity within this group of patients when compared to SLE and SSc, where three rules were identified for each of them. In RA, the association rules most frequently included high expression of *TLR3* and/or *IL1RAP/IL1R3*, thus again highlighting activation of the innate system in active RA. In SLE, a low expression of *IL1RN* and *IL18R1* and in SSc, a low level of *TLR5* and *IL18R1* occurred ofen in the rules. Applying association rules (combinations of genes describing a particular disease), excellent confidence and accuracy above 77% was achieved for all investigated diseases.

Interestingly, about half of the patients in each disease were characterized by multiple rules, while others were typical by only one gene expression pattern rule. The existence of several innate profile subgroups within RA patients lets us suggest that the heterogeneity in the innate pattern in RA may contribute to various clinical disease manifestations [4, 16], thus deserving future investigation. We also hypothesize that observed heterogeneity in the innate signature may contribute to the heterogeneity in the IFN signature recently reported in RA [4]. Our data further highlighted the application of advanced multivariate data analysis especially for diseases such as SLE, where many clinical phenotypes exist. This may be reflected in the high variability in the expression pattern which might be underestimated by univariate statistics, especially in the case of low abundant genes. Finally, our data points out the involvement of various key innate molecules as well as the different interplay between individual innate receptors in the studied diseases.

To gain a more complete picture of the innate signature in autoimmune diseases, we report also the differential profile of the innate signature in studied diseases compared to healthy controls. This comparison revealed the upregulation of four members of TLR (*TLR2*, *TLR3*, *TLR5*, and *TLR8*) and six members of the IL1/IL1R family (*IL1B*, *IL1RN*, *IL1RAP*, *IL18R1*, *IL18*, and *SIGIRR*) in RA when compared to healthy controls. In line with our results, deregulation of these genes or their protein products was already registered in RA [16, 30, 32, 39–44]. In SLE, this study showed for the first time downregulation of *TLR10*, a broad negative regulator of TLR signaling [45, 46]. The first evidence about the possible involvement of TLR10 in autoimmunity has been already observed: downregulated *TLR10* expression was reported in PBMC of patients with microscopic polyangiitis [47] as well as RA patients with active disease [16]. In contrast to the murine models of SLE [48], we did not observe increased *TLR7* and *TLR9* expression in our SLE patients. In SSc, our study revealed upregulation of *IL1RN*, *IL18*, and *CXCL8* and downregulation of *IL1RAP* and *IL18R1*. In line with our results, upregulated *IL1RN* mRNA [49], increased IL18 expression in skin biopsies [50], and elevated serum IL8 in patients with scleroderma [51] were reported. Here, we report for the first time downregulation of *IL1RAP* and *IL18R1* in SSc. IL1RAP (IL1R3) is a coreceptor of IL1R1 and is indispensable for the transmission of IL1 signaling [35]. Regarding *IL18R1*, it encodes the *α* subunit of the IL18 receptor responsible for IL18 binding. The activated receptor then initiates the same signaling pathway as IL1 to activate NF-*κ*B [52]. How these proteins contribute to the SSc pathogenesis deserves future investigations.

The authors are aware of some limitations. The study was performed as a cross-sectional analysis in a real-world setting of patients in different stages of the disease; however, the authors restricted analysis only to patients in the active disease stage in order to obtain a more homogenous cohort. Due to the small number of patients in the subgroups with particular gene patterns revealed by association analysis, the subanalysis of their association with clinical parameters was not performed. Future studies on larger cohorts with well-defined patients would be advisable to further confirm our results.

## 5. Conclusions

To conclude, this first cross-disease study highlighted the heterogeneous nature among and within RA, SLE, and SSc, with the identification of RA having the highest diversity and abundance in the innate signature when compared to SLE and SSc. Moreover, the results from applied data mining approaches show the importance of a multiple multivariate analysis for better understanding of relationships between individual molecules, especially in highly heterogeneous diseases.

## Figures and Tables

**Table 1:** Demographic and clinical characteristics of enrolled patients.

	RA (*n* = 36)	SLE (*n* = 28)	SSc (*n* = 22)
Female/male	26/10	24/4	15/7
Age (years) mean (min-max)	57.5 (39-80)	40.1 (19-67)	58.0 (38-77)
Duration of the disease (years) mean (min-max)	18.1 (9-50)	10.0 (1-20)	5.4 (0-21)
Medications (% (*n*))			
Steroids	89 (32)	82 (23)	96 (21)
NSAIDs	78 (28)	14 (4)	0 (0)
Methotrexate	83 (30)	14 (4)	9 (2)
Other DMARDs^∗^	36 (13)	100 (28)	73 (16)
Biologics	39 (14)	0 (0)	0 (0)
Relative white blood count (%)			
Lymphocytes (mean (95% CI))	24.9 (20.5-29.3)	22.9 (18.5-27.3)	21.4 (17.5-25.4)
Neutrophils (mean (95% CI))	62.9 (57.9-67.9)	67.1 (61.6-72.6)	67.3 (62.5-72.2)
Monocytes (mean (95% CI))	8.9 (7.9-9.9)	8.5 (7.1-9.9)	9.2 (7.9-10.4)

NSAIDs: nonsteroidal anti-inflammatory drugs; DMARDs: disease-modifying antirheumatic drugs; CI: confidence interval. ^∗^Other DMARDs taken were hydroxychloroquine (RA/SLE/SSc; *n* = 3/26/0), leflunomide (8/0/0), sulfasalazine (2/0/0), azathioprine (0/8/12), mycophenolate mofetil (0/6/0), cyclophosphamide (0/3/3), and cyclosporine (0/1/1).

**Table:** (a) RA vs. SLE

Gene	Mean (95% CI)		FC	*P* value	*P*_corr_
	RA	SLE			
*TLR5*	0.056 (0.043-0.070)	0.021 (0.011-0.032)	6.49	5.2 × 10^−4^	9.3 × 10^−3^
*SIGIRR*	0.300 (0.247-0.353)	0.179 (0.141-0.218)	1.76	2.0 × 10^−3^	2.0 × 10^−2^
*TLR2*	0.077 (0.059-0.095)	0.046 (0.029-0.063)	2.00	3.7 × 10^−3^	2.2 × 10^−2^

**Table:** (b) RA vs. SSc

Gene	Mean (95% CI)		FC	*P* value	*P*_corr_
	RA	SSc			
*IL1RAP*	0.015 (0.011-0.020)	0.003 (0.001-0.004)	6.08	1.7 × 10^−7^	3.0 × 10^−6^
*TLR5*	0.056 (0.043-0.070)	0.013 (0.007-0.019)	7.16	1.1 × 10^−5^	9.8 × 10^−5^
*IL18R1*	0.011 (0.008-0.014)	0.003 (0.002-0.005)	4.08	2.0 × 10^−5^	1.2 × 10^−4^
*SIGIRR*	0.300 (0.247-0.353)	0.155 (0.098-0.211)	2.26	5.9 × 10^−4^	2.6 × 10^−3^
*TLR3*	0.005 (0.003-0.007)	0.001 (6.1 × 10^−5^‐0.001)	28.5	1.8 × 10^−3^	6.6 × 10^−3^

**Table:** (c) SSc vs. SLE

Gene	Mean (95% CI)		FC	*P* value	*P*_corr_
	SSc	SLE			
*IL1R1*	0.004 (0.003-0.005)	0.002 (3.1 × 10^−5^‐0.004)	34.8	2.7 × 10^−4^	4.8 × 10^−3^

*P*_corr_ value corrected for multiple comparisons (Benjamini-Hochberg correction). FC (fold change) between group medians of relative mRNA expression levels.

**Table 3:** Association rules identified for (a) RA, (b) SLE, and (c) SSc.

No.	Rule	Support	Confidence	Number of patients identified
(a) RA				
1	*TLR3* high & *IL1RAP* high	0.13	1.00	11
2	*TLR3* high & *TLR10* high	0.12	1.00	10
3	*TLR3* high & *TLR9* low	0.12	1.00	10
4	*TLR4* low & *TLR8* high & *IL1RAP* high	0.14	1.00	12
5	*TLR5* high & *IL18* high & *IL18R1* low	0.14	1.00	12
6	*TLR6* low & *IL1R1* high & *SIGIRR* high & *CXCL8* low	0.12	0.91	10

(b) SLE				
1	*TLR5* low & *TLR6* high & *IL1RN* low & *IL18R1* low	0.10	0.90	9
2	*TLR1* low & *TLR8* low & *IL1R1* low & *IL1RN* low & *IL18* low & *IL18R1* low	0.13	0.85	11
3	*TLR3* low & *IL1B* high	0.10	0.82	9

(c) SSc				
1	*TLR5* low & *IL1RN* high & *IL18R1* low	0.10	1.00	9
2	*TLR5* low & *TLR3* low & *IL18* high	0.10	0.82	9
3	*TLR5* low & *IL1R1* high & *IL18R1* low	0.10	0.75	9

The data set for each gene was divided into low/high expression by means of a particular gene expression of the whole data set.

## Data Availability

The data used to support the findings of this study are available from the corresponding author upon request.

## Supplementary Materials

Supplementary Materials Methods: description of Andrews curves analysis. Correlation analysis of gene patterns in RA, SLE, and SSc. Table S1: investigated genes and primers used for qRT-PCR. Table S2: relative mRNA expression levels of genes differentially expressed between (A) RA vs. healthy controls, (B) SLE vs. healthy controls, and (C) SSc vs. healthy controls. Table S3: relative mRNA expression levels of genes differentially expressed between (A) RA vs. SLE, (B) RA vs. SSc, and (C) SSc vs. SLE. Figure S1: algorithm flow chart of statistics and advanced data-mining methods used in this study. Figure S2: Andrews curve analysis using a gene expression data which does not discriminate between diseases—representative example. Figure S3: Andrews curves using a set of significantly deregulated genes between (A) RA vs. SLE, (B) RA vs. SSc, and (C) SLE vs. SSc. Figure S4: relative mRNA expression levels of genes differentially expressed in (A) RA vs. healthy controls, (B) SLE vs. healthy controls, and (C) SSc vs. healthy controls. Figure S5: Andrews curves using a set of genes revealed by association rules for discrimination of RA, SLE, and SSc. Figure S6: correlation network for studied innate genes in (A) RA, (B) SLE, and (C) SSc. Equation S1: formula used for computing and plotting of Andrews curves.
