# Supplementary material for: Cross-Disease Innate Gene Signature: Emerging Diversity and Abundance in RA Comparing to SLE and SSc
Source: J Immunol Res. 2019 Jul 16;2019:3575803. doi: 10.1155/2019/3575803 (PMC6664489; doi:10.1155/2019/3575803)
Supplement: Supplementary Materials — Methods: description of Andrews curves analysis. Correlation analysis of gene patterns in RA, SLE, and SSc. Table S1: investigated genes and primers used for qRT-PCR. Table S2: relative mRNA expression levels of genes differentially expressed between (A) RA vs. healthy controls, (B) SLE vs. healthy controls, and (C) SSc vs. healthy controls. Table S3: relative mRNA expression levels of genes differentially expressed between (A) RA vs. SLE, (B) RA vs. SSc, and (C) SSc vs. SLE. Figure S1: algorithm flow chart of statistics and advanced data-mining methods used in this study. Figure S2: Andrews curve analysis using a gene expression data which does not discriminate between diseases—representative example. Figure S3: Andrews curves using a set of significantly deregulated genes between (A) RA vs. SLE, (B) RA vs. SSc, and (C) SLE vs. SSc. Figure S4: relative mRNA expression levels of genes differentially expressed in (A) RA vs. healthy controls, (B) SLE vs. healthy controls, and (C) SSc vs. healthy controls. Figure S5: Andrews curves using a set of genes revealed by association rules for discrimination of RA, SLE, and SSc. Figure S6: correlation network for studied innate genes in (A) RA, (B) SLE, and (C) SSc. Equation S1: formula used for computing and plotting of Andrews curves. [file 3575803.f1.pdf]

SUPPLEMENTARY MATERIAL

Cross-disease innate gene signature: emerging diversity and abundance in RA comparing to SLE and SSc

Anna Petrackova, Pavel Horak, Martin Radvansky, Martina Skacelova, Regina Fillerova, Milos Kudelka, Andrea Smrzova, Frantisek Mrazek, Eva Kriegova

1) Description of Andrews curves analysis

Andrews curves analysis was applied for visualization of structure in multidimensional expression data in studied diseases [1, 2, 3, 4]. The curves are calculated and plotted using relative gene expression values which are transformed using Andrews' function (Equation S1). Visualization of Andrews' function is on a closed interval [-π,π], displayed on the x-axis. The transformed relative gene expression values are plotted on the y-axis. Due to the properties of Andrews' function, the function value is meaningless and is used mainly for visualization of multidimensional feature space into two-dimensional space. For computing and plotting, package Andrews from R library was used [5].

Andrews curves provide a plane representation of the multivariate data using orthogonal basis functions that are able to preserve means, distance and variances [1, 3, 4]. Briefly, Andrews curve for each patient is calculated and plotted for a set of transformed gene expression values, each disease group is marked by different color. For each disease group mean and confidence intervals (95CI) of transformed Andrews curve is presented and resulting curves are visualized in the same plot. The result is demonstrated by visualization of curves: disease groups which differ in gene expression patterns of studied genes exhibit separation of Andrews curve's amplitudes and phase shift. The curves of similar observations of relative gene expression, where no difference in expression values for given genes among studied groups were observed, overlap between investigated groups.

The example of non-separated Andrews curves (demonstrating no difference in expression values for given genes among studied groups) is shown in Figure S2. Andrews curves for gene sets that differ in expression patterns between studied diseases are shown in Figure S3.

Equation S1: Formula used for computing and plotting of Andrews curves.

$$f(t) = \frac{1}{\sqrt{2}}\Big(x_1 + x_2\big(sin(t) + cos(t)\big) + x_3\big(sin(t) - cos(t)\big) + x_4\big(sin(2t) + cos(2t)\big) + x_5(\cdots)\Big)$$

The set of patients in study $P = \{X_1, \dots, X_n\}$, n is the number of patients. $X_i = \{x_1, \dots, x_m\}$ where m is number of measured genes, then x_m is the mth gene expression value for particular patient and gene. Plotting parameter $t \in [-\pi, \pi\,]$.

2) Correlation analysis of gene patterns in RA, SLE, and SSc

We constructed correlation networks depicting the most informative genes based on the correlations between the expression levels of the studied innate genes. The correlation network for active RA was characterized by the expression of *TLR2*, *TLR3*, *TLR5*, and *TLR8* (Figure S6A). In active SLE, the expression of *TLR1*, *TLR5*, *TLR8*, *IL18*, and *IL1RN* genes was the most informative (Figure S6B). In active SSc, the most informative was the expression of *TLR2*, *TLR5*, and *IL1RN* genes (Figure S6C). The mentioned genes showed the highest correlation with the other genes in particular diseases, highlighting the *TLR5* gene expression in all studied diseases.

3) Tables and Figures

Table S1: Investigated genes and primers used for qRT-PCR.

Gene symbol	Gene product	RefSeq accession no.	Forward/reverse primer sequence (5’ to 3’)	Amplicon length (bp)
PGK1	Phosphoglycerate kinase 1	NM_000291	CTCAACAACATGGAGATTGG/CTTTGGACATTAGGTCTTTGAC	76
TLR1	Toll-like receptor 1	NM_003263	CCCTACAAAAGGAATCTGTATC/TGCTAGTCATTTTGGAACAC	89
TLR2	Toll-like receptor 2	NM_003264	CTTTCAACTGGTAGTTGTGG/GGAATGGAGTTTAAAGATCCTG	176

TLR3	Toll-like receptor 3	NM_003265	AGATTCAAGGTACATCATGC/CAATTTATGACGAAAGGCAC	195
TLR4	Toll-like receptor 4	NM_138557	GATTTATCCAGGTGTGAAATCC/TATTAAGGTAGAGAGGTGGC	75
TLR5	Toll-like receptor 5	NM_003268	ATCTTTCACATGGGTTTGTC/TTCCCCCAGAAGGTTATATG	170
TLR6	Toll-like receptor 6	NM_006068	CTGCCCAAGATTCAGGAGTG/CCATTGCCTTACAACAAAGTTCT	63
TLR7	Toll-like receptor 7	NM_016562	AGATATAGGATCACTCCATGC/CTTCCAAAATGGAATGTAGAGG	120
TLR8	Toll-like receptor 8	NM_138636	TGGAAAACATGTTCCTTCAG/TGCTTTTTCTCATCACAAGG	121
TLR9	Toll-like receptor 9	NM_017442	AAATCCCTCATATCCCTGTC/TTGTAATAACAGTTGCCGTC	116
TLR10	Toll-like receptor 10	NM_030956	AGATTGCTTTTGCCACCAAC/TCTCACATCTCCTTTTGATAGCC	114
IL1B	Interleukin 1 beta	NM_000576	CTAAACAGATGAAGTGCTCC/GGTCATTCTCCTGGAAGG	183
IL1RN	Interleukin 1 receptor antagonist	NM_173842	ATACTTGCAAGGACCAAATG/TGTTAACTGCCTCCAGC	155
IL18	Interleukin 18	NM_001562	CCTTTAAGGAAATGAATCCTCC/CATCTTATTATCATGTCCTGGG	95
IL1R1	Interleukin 1 receptor type 1	NM_000877	TGTTCATTTATGGAAGGGATGA/TTCTGCTTTTCTTTACGTTTTCATT	78
IL1RAP	Interleukin 1 receptor accessory protein	NM_002182	AACTTGAGTTTCCTCATTGC/AGCCTACTACCTTTACAGTC	121
IL18R1	Interleukin 18 receptor 1	NM_003855	GTGAGAAAAGCAGACATGG/AAATGACACACACAGTCAC	112
SIGIRR/ *IL1R8*	Single Ig and TIR domain containing	NM_001135054	ACCCATCTTCATCACCTTC/AAAATCGGAGGAAGGAGTC	133
CXCL8/ IL8	C-X-C motif chemokine ligand 8	NM_000584	GGCACAAACTTTCAGAGACAG/ACACAGAGCTGCAGAAATCAG	153

Table S2: Relative mRNA expression levels of genes differentially expressed between A) RA *vs* healthy controls, B) SLE *vs* healthy controls, and C) SSc *vs* healthy controls.

A) RA *vs* healthy controls

Gene	*Mean (95 % CI)*		*FC*	*P*	P_{corr}
	Healthy controls	*RA*			
TLR5	0.026 (0.018-0.033)	0.056 (0.043-0.070)	3.85	0.0001	0.002
IL18R1	0.005 (0.004-0.007)	0.011 (0.008-0.014)	1.98	0.0003	0.002
IL1RN	0.019 (0.013-0.025)	0.039 (0.030-0.047)	3.01	0.0003	0.002
IL1RAP	0.008 (0.006-0.010)	0.015 (0.011-0.020)	2.43	0.0004	0.002
SIGIRR	0.188 (0.159-0.216)	0.300 (0.247-0.353)	1.61	0.0004	0.002
IL18	0.037 (0.031-0.042)	0.054 (0.044-0.064)	1.54	0.0005	0.002
TLR8	0.039 (0.032-0.047)	0.061 (0.049-0.072)	1.98	0.001	0.003
TLR2	0.050 (0.038-0.062)	0.077 (0.059-0.095)	1.94	0.004	0.01
IL1B	0.035 (0.006-0.064)	0.044 (0.028-0.061)	2.49	0.02	0.03
TLR3	0.003 (0.002-0.003)	0.005 (0.003-0.007)	4.60	0.03	0.05
IL1R1	0.003 (0.002-0.003)	0.005 (0.003-0.006)	1.63	0.05	>0.05
CXCL8	0.117 (0.043-0.192)	0.112 (0.055-0.170)	2.43	>0.05	>0.05
TLR1	0.054 (0.045-0.062)	0.061 (0.051-0.071)	1.17	>0.05	>0.05
TLR4	0.051 (0.045-0.058)	0.046 (0.037-0.055)	1.03	>0.05	>0.05
TLR6	0.032 (0.022-0.042)	0.030 (0.024-0.035)	1.11	>0.05	>0.05
TLR9	0.009 (0.008-0.011)	0.009 (0.007-0.011)	0.87	>0.05	>0.05
TLR10	0.007 (0.006-0.008)	0.007 (0.005-0.010)	0.92	>0.05	>0.05
TLR7	0.015 (0.013-0.017)	0.015 (0.012-0.018)	1.07	>0.05	>0.05

B) SLE *vs* healthy controls

Gene	*Mean (95 % CI)*		*FC*	*P*	P_{corr}
	Healthy controls	*SLE*			
TLR10	0.008 (0.006-0.010)	0.007 (0.002-0.012)	0.34	0.02	>0.05
TLR4	0.044 (0.034-0.054)	0.084 (0.044-0.125)	1.33	>0.05	>0.05
IL18R1	0.004 (0.003-0.006)	0.008 (0.004-0.012)	2.81	>0.05	>0.05
IL1R1	0.002 (0.001-0.003)	0.002 (0.000-0.005)	0.08	>0.05	>0.05
SIGIRR	0.223 (0.185-0.261)	0.179 (0.141-0.218)	0.79	>0.05	>0.05
CXCL8	0.154 (0.017-0.291)	0.237 (0.104-0.371)	1.98	>0.05	>0.05
TLR6	0.030 (0.022-0.038)	0.045 (0.031-0.060)	1.20	>0.05	>0.05
TLR9	0.010 (0.008-0.012)	0.011 (0.006-0.016)	0.73	>0.05	>0.05
TLR3	0.002 (0.001-0.003)	0.001 (0.000-0.002)	0.48	>0.05	>0.05
TLR2	0.040 (0.023-0.057)	0.046 (0.029-0.063)	1.82	>0.05	>0.05
IL1B	0.051 (0.000-0.106)	0.045 (0.018-0.071)	0.22	>0.05	>0.05
IL1RAP	0.010 (0.005-0.014)	0.022 (0.005-0.040)	0.08	>0.05	>0.05
IL1RN	0.022 (0.014-0.030)	0.042 (0.015-0.069)	1.02	>0.05	>0.05
TLR1	0.048 (0.037-0.059)	0.055 (0.039-0.071)	0.83	>0.05	>0.05
TLR5	0.021 (0.013-0.030)	0.021 (0.011-0.032)	0.59	>0.05	>0.05
IL18	0.039 (0.033-0.045)	0.041 (0.033-0.049)	0.87	>0.05	>0.05
TLR7	0.014 (0.011-0.018)	0.018 (0.010-0.026)	1.01	>0.05	>0.05
TLR8	0.044 (0.030-0.058)	0.050 (0.029-0.071)	1.03	>0.05	>0.05

C) SSc *vs* healthy controls

Gene	*Mean (95 % CI)*	*FC*	*P*	P_{corr}

	Healthy controls	*SSc*			
IL1RN	0.019 (0.012-0.026)	0.037 (0.026-0.047)	2.78	0.001	0.01
IL1RAP	0.007 (0.005-0.009)	0.003 (0.001-0.004)	0.35	0.002	0.01
CXCL8	0.084 (0.039-0.130)	0.110 (0.062-0.159)	3.09	0.003	0.02
IL18R1	0.006 (0.005-0.007)	0.003 (0.001-0.005)	0.38	0.01	0.04
IL18	0.036 (0.029-0.042)	0.047 (0.039-0.055)	1.40	0.01	0.05
TLR3	0.003 (0.002-0.004)	0.001 (0.000-0.001)	0.16	0.05	>0.05
TLR8	0.036 (0.028-0.044)	0.047 (0.035-0.058)	1.47	>0.05	>0.05
IL1R1	0.003 (0.002-0.004)	0.004 (0.003-0.005)	1.30	>0.05	>0.05
SIGIRR	0.195 (0.160-0.229)	0.155 (0.098-0.211)	0.70	>0.05	>0.05
TLR5	0.026 (0.017-0.035)	0.013 (0.007-0.018)	0.61	>0.05	>0.05
IL1B	0.036 (0.000-0.073)	0.027 (0.001-0.053)	1.59	>0.05	>0.05
TLR10	0.006 (0.005-0.008)	0.005 (0.003-0.007)	1.04	>0.05	>0.05
TLR7	0.014 (0.012-0.017)	0.013 (0.010-0.015)	0.86	>0.05	>0.05
TLR1	0.057 (0.046-0.067)	0.049 (0.041-0.057)	0.91	>0.05	>0.05
TLR6	0.025 (0.020-0.030)	0.025 (0.021-0.028)	1.14	>0.05	>0.05
TLR4	0.052 (0.045-0.058)	0.054 (0.045-0.063)	1.20	>0.05	>0.05
TLR9	0.009 (0.007-0.011)	0.010 (0.007-0.013)	1.08	>0.05	>0.05
TLR2	0.052 (0.038-0.065)	0.048 (0.035-0.062)	1.00	>0.05	>0.05

P_{corr} value corrected for multiple comparisons (Benjamini-Hochberg correction)

FC (Fold Change) between group medians of relative mRNA expression levels.

Table S3: Relative mRNA expression levels of genes differentially expressed between A) RA *vs* SLE, B) RA *vs* SSc, and C) SSc *vs* SLE.

A) RA *vs* SLE

Gene	*Mean (95 % CI)*		*FC*	*P*	P_{corr}
	RA	*SLE*			
TLR5	0.056 (0.043-0.070)	0.021 (0.011-0.032)	6.49	0.0005	0.01
SIGIRR	0.300 (0.247-0.353)	0.179 (0.141-0.218)	1.76	0.002	0.02
TLR2	0.077 (0.059-0.095)	0.046 (0.029-0.063)	2.00	0.004	0.02
IL18	0.054 (0.044-0.064)	0.041 (0.033-0.049)	1.45	0.02	>0.05
TLR3	0.005 (0.003-0.007)	0.001 (0.000-0.002)	15.5	0.02	>0.05
IL1R1	0.005 (0.003-0.006)	0.002 (0.000-0.005)	41.5	0.02	>0.05
TLR8	0.061 (0.049-0.072)	0.050 (0.029-0.071)	2.13	0.03	>0.05
IL18R1	0.011 (0.008-0.014)	0.008 (0.004-0.012)	1.46	0.04	>0.05
IL1RAP	0.015 (0.011-0.020)	0.022 (0.005-0.040)	37.0	0.04	>0.05
IL1RN	0.039 (0.030-0.047)	0.042 (0.015-0.069)	2.66	0.05	>0.05
IL1B	0.044 (0.028-0.061)	0.045 (0.018-0.071)	6.26	>0.05	>0.05
TLR1	0.061 (0.051-0.071)	0.055 (0.039-0.071)	1.45	>0.05	>0.05
CXCL8	0.112 (0.055-0.170)	0.237 (0.104-0.371)	0.60	>0.05	>0.05
TLR6	0.030 (0.024-0.035)	0.045 (0.031-0.060)	0.80	>0.05	>0.05
TLR10	0.007 (0.005-0.010)	0.007 (0.002-0.012)	1.71	>0.05	>0.05
TLR4	0.046 (0.037-0.055)	0.084 (0.044-0.125)	0.99	>0.05	>0.05
TLR7	0.015 (0.012-0.018)	0.018 (0.010-0.026)	1.21	>0.05	>0.05
TLR9	0.009 (0.007-0.011)	0.011 (0.006-0.016)	0.97	>0.05	>0.05

B) RA *vs* SSc

Gene	*Mean (95 % CI)*		*FC*	*P*	P_{corr}
	RA	*SSc*			
IL1RAP	0.015 (0.011-0.020)	0.003 (0.001-0.004)	6.08	1.7×10^{-7}	3.0×10^{-6}
TLR5	0.056 (0.043-0.070)	0.013 (0.007-0.019)	7.16	1.1×10^{-5}	9.8×10^{-5}
IL18R1	0.011 (0.008-0.014)	0.003 (0.002-0.005)	4.08	2.0×10^{-5}	0.0001
SIGIRR	0.300 (0.247-0.353)	0.155 (0.098-0.211)	2.26	0.001	0.003
TLR3	0.005 (0.003-0.007)	0.001 (0.000-0.001)	28.5	0.002	0.007
TLR2	0.077 (0.059-0.095)	0.048 (0.035-0.062)	1.87	0.02	>0.05
TLR4	0.046 (0.037-0.055)	0.054 (0.045-0.063)	0.84	0.05	>0.05
TLR8	0.061 (0.049-0.072)	0.047 (0.035-0.058)	1.55	>0.05	>0.05
TLR1	0.061 (0.051-0.071)	0.049 (0.041-0.057)	1.25	>0.05	>0.05
IL1B	0.044 (0.028-0.061)	0.027 (0.001-0.053)	1.67	>0.05	>0.05
TLR7	0.015 (0.012-0.018)	0.013 (0.010-0.015)	1.26	>0.05	>0.05
CXCL8	0.112 (0.055-0.170)	0.110 (0.062-0.159)	0.99	>0.05	>0.05
TLR10	0.007 (0.005-0.010)	0.005 (0.003-0.007)	0.97	>0.05	>0.05
IL18	0.054 (0.044-0.064)	0.047 (0.039-0.055)	1.17	>0.05	>0.05
TLR6	0.030 (0.024-0.035)	0.025 (0.021-0.028)	1.02	>0.05	>0.05
TLR9	0.009 (0.007-0.011)	0.010 (0.007-0.013)	0.95	>0.05	>0.05
IL1RN	0.039 (0.030-0.047)	0.037 (0.026-0.047)	1.04	>0.05	>0.05
IL1R1	0.005 (0.003-0.006)	0.004 (0.003-0.005)	1.19	>0.05	>0.05

C) SSc *vs* SLE

Gene	*Mean (95 % CI)*	*FC*	*P*	P_{corr}

	SSc	*SLE*			
IL1R1	0.004 (0.003-0.005)	0.002 (0.000-0.005)	34.8	0.0003	0.005
IL18R1	0.003 (0.002-0.005)	0.008 (0.004-0.012)	0.36	0.01	>0.05
IL1RN	0.037 (0.026-0.047)	0.042 (0.015-0.069)	2.56	>0.05	>0.05
IL18	0.047 (0.039-0.055)	0.041 (0.033-0.049)	1.24	>0.05	>0.05
TLR6	0.025 (0.021-0.028)	0.045 (0.031-0.060)	0.78	>0.05	>0.05
IL1RAP	0.003 (0.001-0.004)	0.022 (0.005-0.040)	6.08	>0.05	>0.05
TLR3	0.001 (0.000-0.001)	0.001 (0.000-0.002)	0.54	>0.05	>0.05
TLR8	0.047 (0.035-0.058)	0.050 (0.029-0.071)	1.37	>0.05	>0.05
SIGIRR	0.155 (0.098-0.211)	0.179 (0.141-0.218)	0.78	>0.05	>0.05
TLR5	0.013 (0.007-0.019)	0.021 (0.011-0.032)	0.91	>0.05	>0.05
TLR2	0.048 (0.035-0.062)	0.046 (0.029-0.063)	1.07	>0.05	>0.05
CXCL8	0.110 (0.062-0.159)	0.237 (0.104-0.371)	0.60	>0.05	>0.05
TLR4	0.054 (0.045-0.063)	0.084 (0.044-0.125)	1.18	>0.05	>0.05
IL1B	0.027 (0.001-0.053)	0.045 (0.018-0.071)	3.75	>0.05	>0.05
TLR1	0.049 (0.041-0.057)	0.055 (0.039-0.071)	1.16	>0.05	>0.05
TLR7	0.013 (0.010-0.015)	0.018 (0.010-0.026)	0.96	>0.05	>0.05
TLR9	0.010 (0.007-0.013)	0.011 (0.006-0.016)	1.02	>0.05	>0.05
TLR10	0.005 (0.003-0.007)	0.007 (0.002-0.012)	1.77	>0.05	>0.05

P_{corr} value corrected for multiple comparisons (Benjamini-Hochberg correction)

FC (Fold Change) between group medians of relative mRNA expression levels.

Figure S1: Algorithm flow chart of statistics and advanced data-mining methods used in this study.

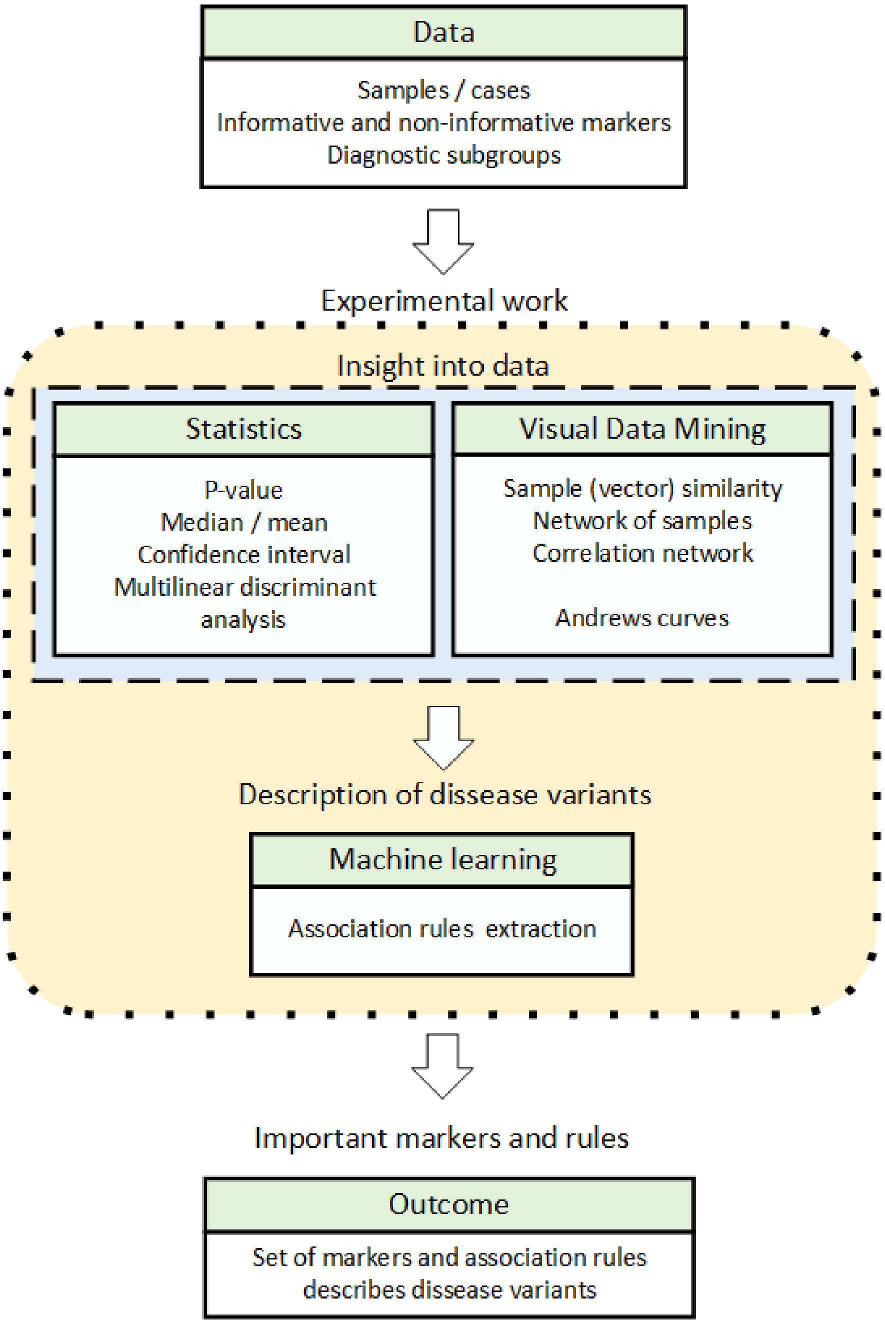

Figure S2: Andrews curves analysis using a gene expression data which does not discriminate between diseases - representative example.

Andrews curves for combination of genes with no differences in their expression signatures between RA and SLE overlap.

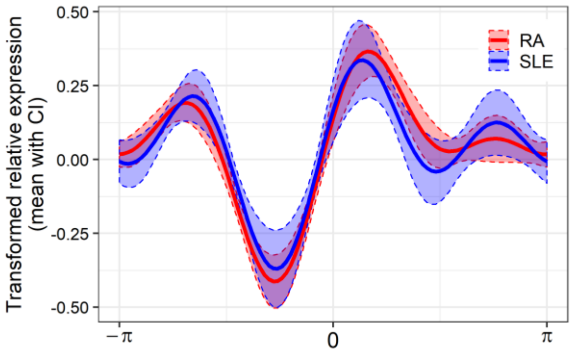

Figure S3: Andrews curves using a set of significantly deregulated genes between A) RA vs SLE; B) RA vs SSc; C) SLE vs SSc.

Upper part shows the Andrews curves as differential transformed relative innate gene expression for particular diseases (RA red, SLE blue, and SSc violet), where the difference is exhibited by separation of curve's amplitudes and phase shift. Andrews curve of similar gene expression signatures in studied diseases overlap, while dissimilar observations exhibit by separation of curve's, preserving means, distances and variances.

The right part shows the combination of the most differentially expressed genes between particular diseases. Full lines represent the mean values, the dashed lines 95% confidence intervals.

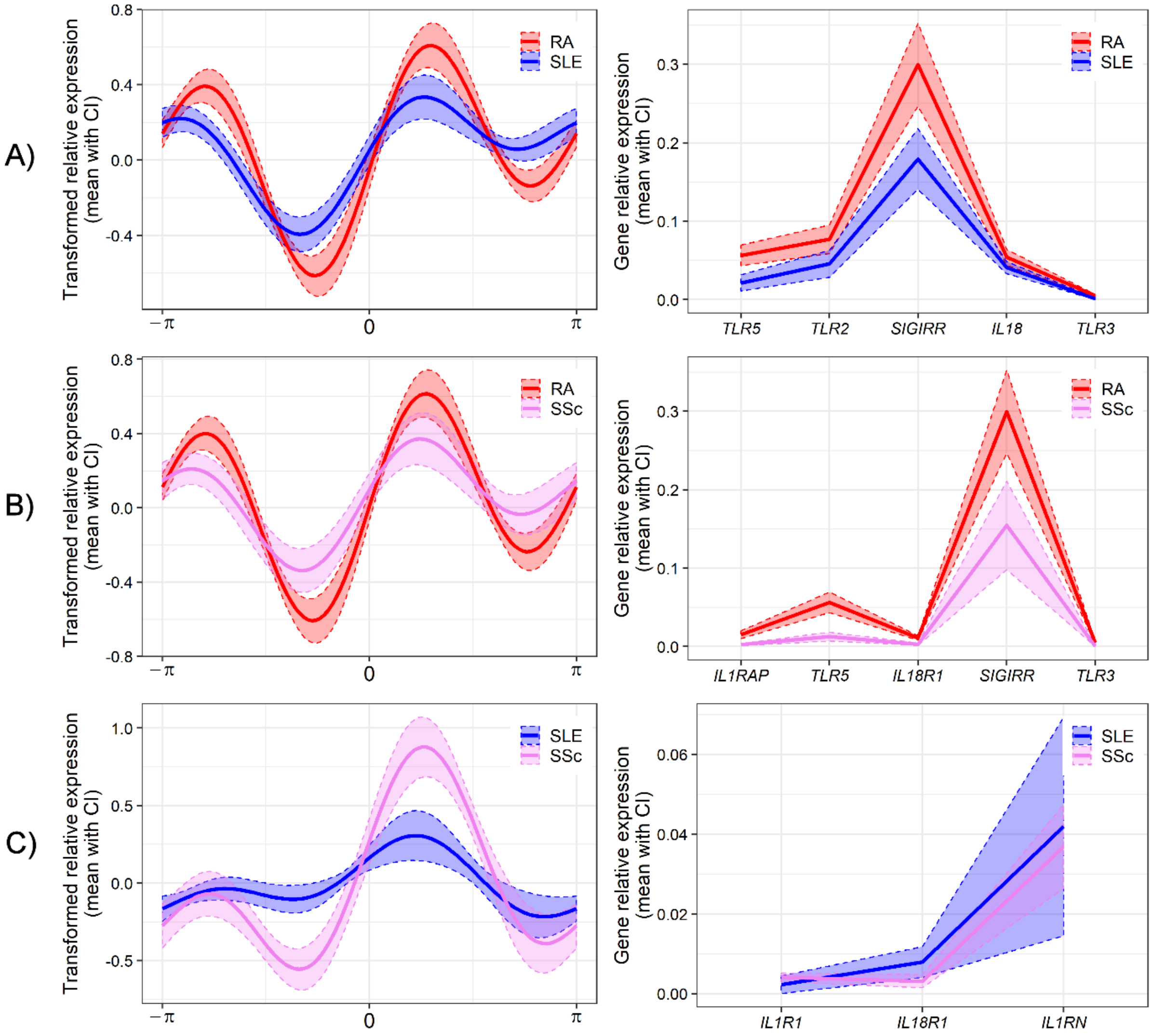

Figure S4: Relative mRNA expression levels of genes differentially expressed in A) RA *vs* healthy controls, B) SLE *vs* healthy controls, and C) SSc *vs* healthy controls.

Group means are indicated by horizontal bars, error bars indicate 95 % CI.

A)

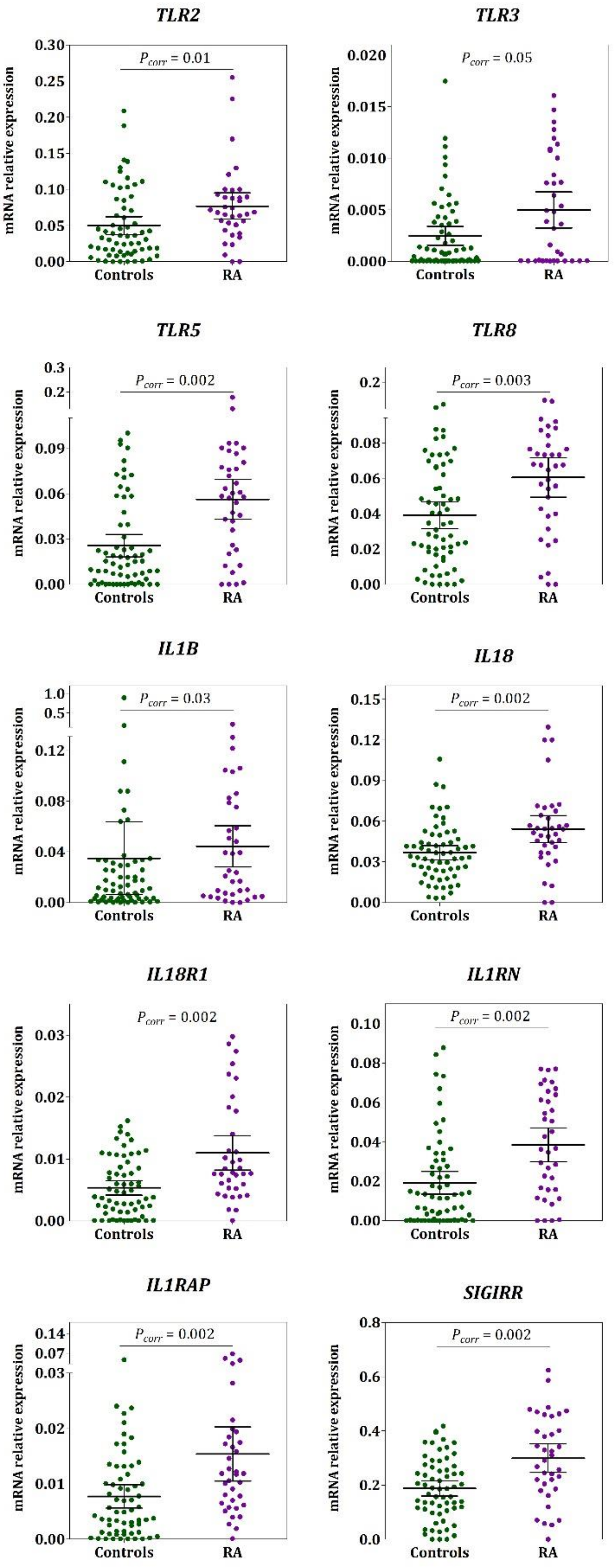

B)

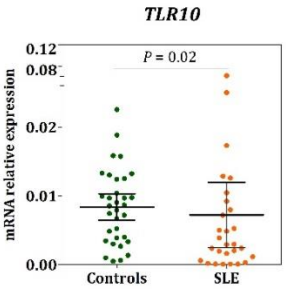
TLR10
P = 0.02
mRNA relative expression
0.12
0.08
0.02
0.01
0.00
Controls
SLE

C)

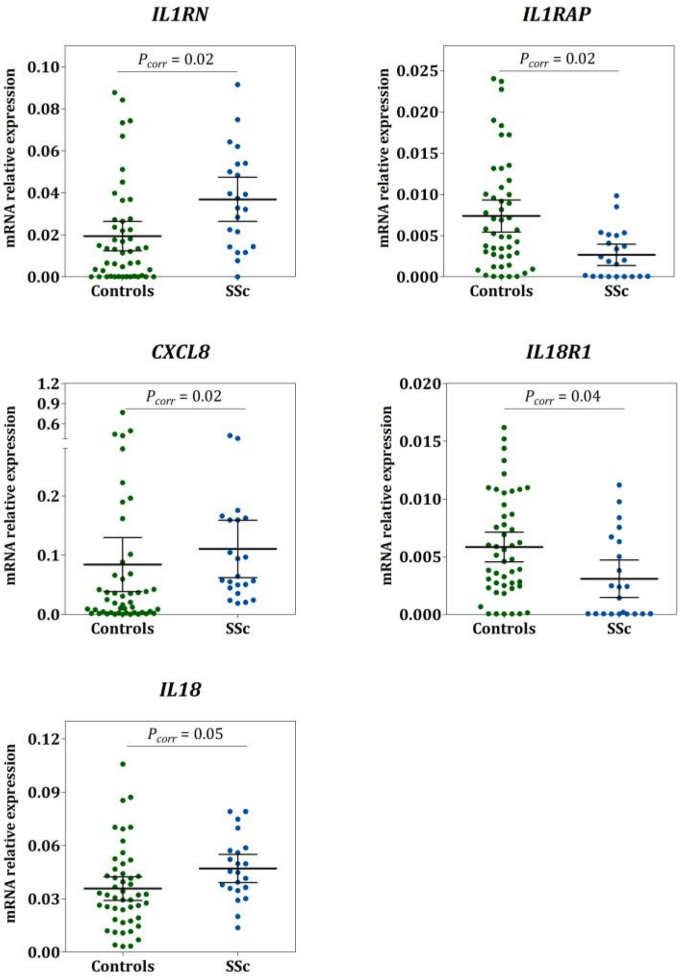
IL1RN
Pcorr = 0.02
mRNA relative expression
0.10
0.08
0.06
0.04
0.02
0.00
Controls
SSc
IL1RAP
Pcorr = 0.02
mRNA relative expression
0.025
0.020
0.015
0.010
0.005
0.000
Controls
SSc
CXCL8
Pcorr = 0.02
mRNA relative expression
1.2
0.9
0.6
0.2
0.1
0.0
Controls
SSc
IL18R1
Pcorr = 0.04
mRNA relative expression
0.020
0.015
0.010
0.005
0.000
Controls
SSc
IL18
Pcorr = 0.05
mRNA relative expression
0.12
0.09
0.06
0.03
0.00
Controls
SSc

Figure S5: Andrews curves using a set of genes revealed by association rules for discrimination of RA, SLE, and SSc.

Andrews curves were plotted for the set of genes (*TLR3*, *TLR5*, *IL18*, *IL18R1*, and *IL1R1*) revealed by association rules; only genes occurring in rules for all studied diseases were used. Andrews curves demonstrates good discriminant power among studied autoimmune diseases. Full lines represent the mean values, the dashed lines 95% confidence intervals.

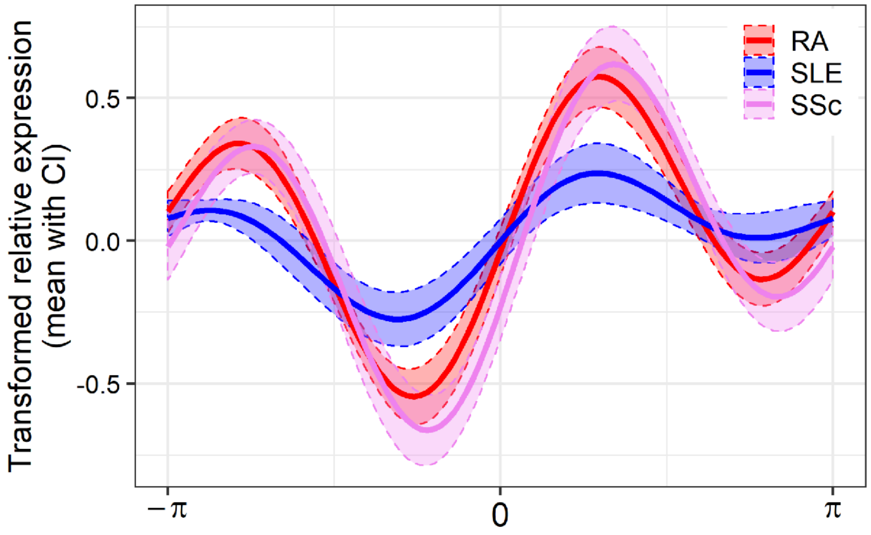

Figure S6: Correlation network for studied innate genes in A) RA, B) SLE, and C) SSc. The size of the nodes represents the representativeness of the particular gene within the innate gene signature for particular disease, the links between genes the correlation power.

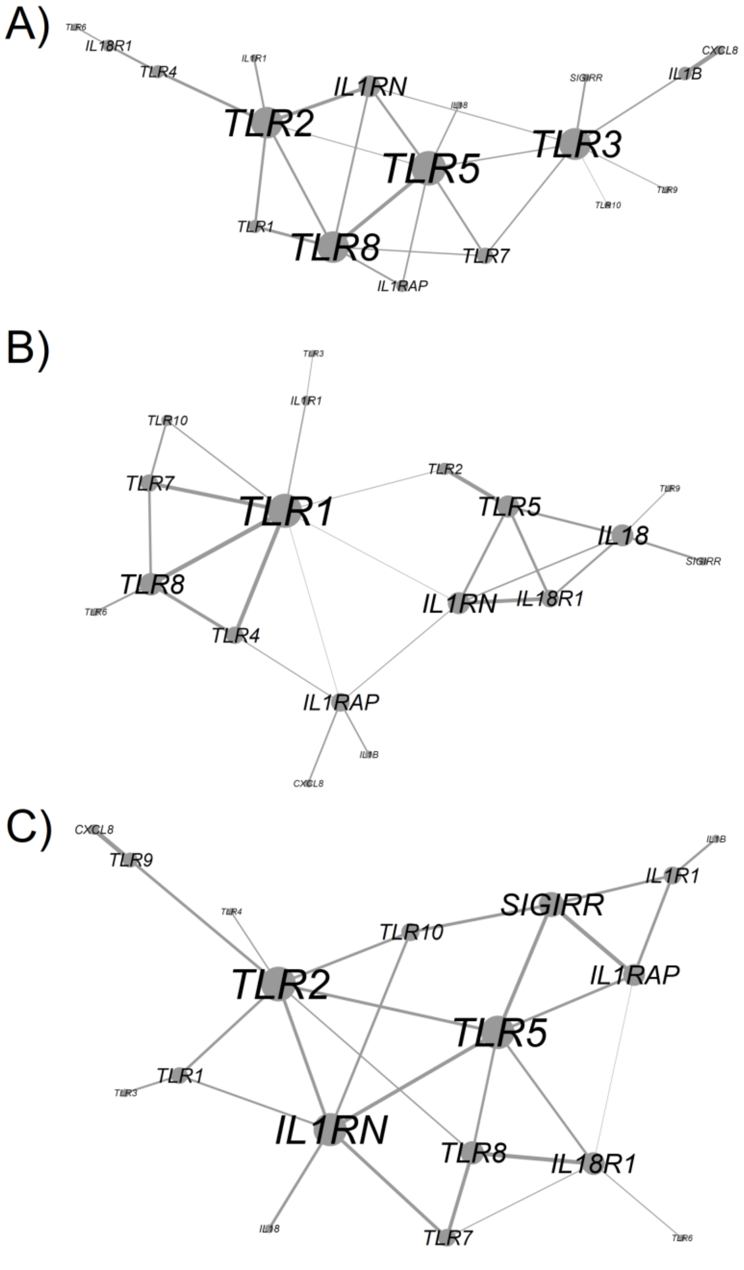

References

[1] Moustafa RE. Andrews curves. WIREs Comp Stat. 2011;3:373-82.

[2] Niedzielski P, Mleczek M, Budka A, Rzymski P, Siwulski M, Jasinska A, et al. A screening study of elemental composition in 12 marketable mushroom species accessible in Poland. Eur Food Res Technol. 2017;243:1759.

[3] Andrews DF. Plots of high-dimensional data. Biometrics. 1972;28:125-36.

[4] García-Osorio C, Fyfe C. Visualization of high-dimensional data via orthogonal curves. J. UCS. 2005;11:1806-19.

[5] Myslivec J. 2012. andrews: Andrews curves. R package version 1.0. http://CRAN.R-project.org/package=andrews
